# AI-Generated Treatment Recommendations in Newly Diagnosed Lung Cancer: A Single-Center Feasibility Study in a Resource-Limited Setting

**DOI:** 10.3390/curroncol33100640

**Published:** 2026-10-21

**Authors:** Andrei Popescu, Mara Jidveian-Popescu, Beatrice Mahler, Rodica Olteanu, Emanuel Clem

**Affiliations:** 1 Department of Medical Oncology, Colentina Clinical Hospital, 020125 Bucharest, Romania; 2 Faculty of Medicine, Titu Maiorescu University, 031593 Bucharest, Romania; directorm@spitalulcolentina.ro (R.O.); emanuel.clem@s.utm.ro (E.C.); 3 Department of Medical Oncology, CF2 Clinical Hospital, 011464 Bucharest, Romania; oncologie@spcf2.ro; 4 Department of Pneumology, “Marius Nasta” Institute of Pneumology, 050159 Bucharest, Romania; beatrice.mahler@umfcd.ro; 5 Department of Pulmonology, “Carol Davila” University of Medicine and Pharmacy, 020021 Bucharest, Romania; 6 Department of Dermatology, Colentina Clinical Hospital, 020125 Bucharest, Romania

**Keywords:** large language models, lung cancer, clinical decision support, multidisciplinary team, artificial intelligence, oncology

## Abstract

Background: How treatment recommendations generated by large language models (LLMs) compare with real-world decisions in oncology remains poorly characterized, particularly in resource-limited settings. Methods: In this single-center feasibility study, four LLMs (ChatGPT, Claude, Gemini, and Grok, accessed via consumer interfaces in June 2026) generated treatment recommendations for 27 consecutive newly diagnosed lung cancer cases treated at a Romanian oncology unit (June 2024–May 2026). Each model’s output and the treatment actually delivered were formatted identically (135 recommendations total), presented in randomized order, and scored for appropriateness (1–5) by a reviewer blinded to the source, with three quality flags. A second independent reviewer re-scored all recommendations to assess inter-rater reliability. Scores were compared using the Friedman test. Results: Mean appropriateness was highest for the real-world decision (4.52), followed by ChatGPT (4.48), Claude (4.44), Grok (4.41), and Gemini (4.37), with no significant difference (Friedman χ^2^_4_ = 1.99, *p* = 0.74). No recommendation scored below 3. Inter-rater agreement was high in raw terms (93.3% within one point) but only fair after chance correction (weighted κ = 0.26), reflecting a ceiling effect. Conclusions: AI-generated recommendations were rated similarly to real-world decisions. The evaluation method proved feasible in a resource-limited setting; the small sample and ceiling effect preclude equivalence claims, and the findings are hypothesis-generating.

## Simple Summary

Artificial intelligence tools such as ChatGPT are being explored to help doctors choose cancer treatments, but almost all of this research comes from large, well-funded hospitals. Whether these tools can be evaluated rigorously in small, resource-limited settings, where they might actually be needed most, is far less clear. In a single Romanian oncology unit, we asked four widely available AI systems to recommend treatments for 27 patients with newly diagnosed lung cancer and compared their suggestions with the treatment each patient actually received. Two independent specialists, unaware of which recommendation came from which source, rated every recommendation for appropriateness. AI-generated and real-world recommendations received similarly high ratings, though the study was too small to prove they are equivalent. Our main finding is that this kind of structured, blinded evaluation is feasible even with very limited resources, providing a practical model for other small centers to assess AI tools before trusting them.

## 1. Introduction

Lung cancer remains the leading cause of cancer death worldwide [1], and its management is among the most complex in oncology. In recent years, large language models (LLMs) have been studied as decision support tools in medicine and oncology, where they can generate structured, guideline-oriented treatment recommendations [2,3,4]. Lung cancer is a demanding test case for such tools: treatment decisions depend on multiple variables, such as stage, histology, molecular profile and performance status, and standards are rapidly evolving with the expansion of immunotherapy and targeted therapies. These decisions are usually made in multidisciplinary team (MDT) meetings, where these complex sets of information are sometimes incomplete and need to be reconciled with the guidelines. A question naturally forms in the mind of clinicians: can modern AI assist with this process?

LLM-generated recommendations have been compared with multidisciplinary team (MDT) decisions across several tumor types, including melanoma [5], primary liver cancers [6] and breast [7], with concordance varying by model and tumor type. More broadly, general-purpose chatbots have been shown to produce cancer treatment recommendations that are frequently, though not always, concordant with guidelines [8], and LLMs have been evaluated as decision support tools in precision oncology, where they can propose reasonable systemic therapy options but also generate recommendations a molecular tumor board would not endorse [9]. In lung cancer, agreement between AI and MDT decisions has been examined for curated decision support systems such as Watson for Oncology [10], but generative LLMs, which produce free-text reasoning rather than selecting from predefined options, remain less studied in this setting. Moreover, most existing studies treat the MDT decision as the reference standard, so the human comparator is not itself scored [5,6]. Notably, almost all of this evidence originates from large, well-resourced academic centers. Whether such tools can be rigorously evaluated in smaller, resource-limited settings, where specialist expertise is scarcer, molecular testing is less consistently available, and AI decision support might therefore offer the greatest benefit, remains largely unexamined [7,11]. This gap has also been noted in broader syntheses of the field [2], yet few studies address it directly.

Our objective was to assess the appropriateness of AI-generated treatment recommendations relative to real-world treatment decisions in newly diagnosed lung cancer and to test the feasibility of this evaluation method. We therefore conducted a feasibility study comparing treatment recommendations generated by four LLMs with real-world treatment decisions in consecutive newly diagnosed lung cancer cases, all scored for appropriateness by two reviewers blinded to the source.

## 2. Methods

We designed a retrospective, single-center feasibility study, in which we included 27 lung cancer cases, both small-cell and non-small-cell, from the Oncology (outpatient) Department of the Colentina Clinical Hospital in Bucharest, Romania, over a period of approximately two years, from June 2024 to May 2026. The cases were selected from a local database of newly diagnosed patients, treatment-naïve for the index malignancy, who received active treatment in our clinic. All consecutive cases meeting this criterion were included; none were excluded. The study was approved by the Colentina Hospital Ethics Committee (Approval no. 23/10 June 2026). All patient data were anonymized; no direct identifiers were retained; only sex, year of birth and month of treatment decision were recorded. The case summaries subsequently submitted to the models contained no names, dates of birth, hospital or record numbers, free-text narrative, or other direct or indirect identifiers; because the submitted material contained no personal data, its use did not entail disclosure of patient-identifiable information and was covered by the ethics approval. We nonetheless note that consumer AI interfaces may retain user inputs and emphasize that no patient-identifiable data were at any point uploaded—a routine data privacy consideration for centers contemplating similar evaluations.

Data were extracted from the physical files of the patients in the department archive (no electronic health record system is in use at our institution) into a structured paper abstraction form by two extractors, one senior oncologist and one medical student. The data abstracted by the student were verified by the senior oncologist. The captured fields were: demographics, ECOG performance status, comorbidities and contraindications, histology, TNM stage and disease extent, biomarker status (PD-L1 and molecular panel), organ function, symptoms, prior treatment, and local access/reimbursement context. Missing fields were presented identically to all sources. A digital case summary template was then generated with AI assistance (as was the abstraction form), and the clinical content was entered manually by the senior oncologist for each case into 27 corresponding files. No patient data were processed by AI at this stage, and all case summaries were finalized before any model was queried.

A standardized prompt was developed to elicit treatment recommendations from each model in a fixed format based on the case summary. This prompt was pilot-tested on two hypothetical cases before finalization. It instructed AI to adhere to a strict format comprising treatment intent, preferred systemic treatment, local/supportive treatment, acceptable alternatives, missing information that could change the decision, key reason and major concern. Responses were capped at 120 words, and the models were instructed to be concise, not invent missing data and not use disclaimers or identify themselves as AI. The full prompt is provided in File S1.

Four commercially available LLMs were queried: ChatGPT (GPT-5.5; OpenAI, San Francisco, CA, USA), Claude (Sonnet 4.6; Anthropic, San Francisco, CA, USA), Gemini (3.5 Flash; Google LLC, Mountain View, CA, USA) and Grok (“Fast” mode, no version number displayed; xAI, Palo Alto, CA, USA; https://grok.com, accessed on 27 June 2026). All were accessed through their consumer chat interfaces on a Windows 11 (Microsoft Corporation, Redmond, WA, USA) desktop using the Microsoft Edge browser, under paid subscriptions (ChatGPT Plus, Claude Pro, Google AI Pro and Grok Premium, respectively); no API access, model fine-tuning or custom generation parameters were used. The model identifiers displayed by each interface at the time of use were: ChatGPT: “Medium” within the GPT-5.5 selector, Claude: Sonnet 4.6 “Low”, Gemini 3.5 Flash and Grok Fast. ChatGPT and Claude were queried on 25 June 2026; Gemini and Grok were queried on 27 June 2026. Default settings were used throughout, including web browsing when enabled by default. The prompt was entered by copy/paste, and the case file was entered by drag and drop in the chat field. Each case was submitted in a new chat session with one exception: case LCAI-02 was entered in Claude in an existing session. Responses were not regenerated, with the accidental exception of case LCAI-14—Gemini, where only the first response was used. A personalization setting affecting response style was inadvertently active for ChatGPT from the start of response collection and was reset to default after case LCAI-18. The stylistic artifact was present only in the output for that case; it was removed, and the clinical content was not affected.

The next step was generating the real-world comparator for each case. This was extracted from the paper abstraction form, which was derived from the treatment actually delivered to the patients. Because these decisions were recorded in routine clinical notes rather than in a structured format (no MDT file was available), they were transcribed by the senior oncologist into the same seven-field structure required of the AI models so that all treatment recommendations could be presented to the reviewer in identical form. Clinical content regarding treatment intent, systemic treatment, local/supportive treatment and key reason was taken from the documented decision and its recorded rationale; no treatment content was added. The fields concerning “acceptable alternatives”, “missing information that could change decision”, and “major concern” are prospective in nature and were not available in routine clinical notes; these were completed by the senior oncologist on the basis of the clinical information available in the file at the time of the original decision. This process was performed after all AI model outputs had been collected.

For each case, the order of the five treatment recommendations was randomized independently using ChatGPT-assisted randomization to minimize pattern-seeking by the reviewer. Each reviewer was blinded as to which model generated the recommendations and also as to which recommendation was the real-world one but was aware that each case contained exactly one real-world decision among the five. A ready-to-print PDF batch was generated for each case, containing, in order: the case summary (recto) followed by a blank verso, then each of the five randomized recommendations (recto) with the reviewer instructions and score card on the verso.

Appropriateness and guideline concordance were judged against international guidelines current at the time of assessment (June 2026): the NCCN Clinical Practice Guidelines in Oncology for non-small-cell lung cancer and for small-cell lung cancer [12,13], and, for oncogene-addicted non-small-cell lung cancer, the ESMO recommendations [14]. These were applied according to each reviewer’s clinical expertise and reflected contemporary standards, including the recent shift toward perioperative immunotherapy and expanded targeted therapy in non-small-cell lung cancer [15]. No single guideline version was prescribed to the reviewers; both applied the standards current in their practice at the time of scoring.

To assess inter-rater reliability, all 135 recommendations were subsequently re-scored by a second, independent reviewer (CȘS), a senior oncologist at the same institution as the cases, who had not participated in treating any of the included cases. This reviewer used the identical instrument and randomized packets, was blinded to the source of each recommendation, and was not shown the first reviewer’s scores. Because this reviewer practices at the same center from which the cases originated, familiarity with local practice patterns cannot be fully excluded; however, he did not participate in treating any of the included cases or in any case presentation.

Statistical analysis was performed in R version 4.6.1 (R Foundation for Statistical Computing, Vienna, Austria) via Posit Cloud (Posit PBC, Boston, MA, USA; https://posit.cloud). Appropriateness scores from the primary (MJ-P) reviewer were compared across all five sources using the Friedman rank-sum test, with each case treated as a block; a *p*-value below 0.05 was considered significant. The three flags were summarized descriptively as proportions per source, without formal between-source testing, given the small sample. As a sensitivity analysis, the association between recommendation length (as word count) and appropriateness scores was assessed using Spearman’s rank correlation. No formal sample size calculation was performed; all 27 consecutive cases were included, consistent with a pilot design. Each case was queried once per model, so intra-model response variability was not assessed. Inter-rater reliability between the two reviewers was assessed using weighted Cohen’s κ (quadratic weights), together with exact and within-one-point percent agreement. The analysis code is provided in File S4.

The study is reported in accordance with the CHART (Chatbot Assessment Reporting Tool) statement [16] (File S3).

## 3. Results

The 27 cases comprised 21 non-small-cell (77.8%) and six small-cell (22.2%); adenocarcinoma was the most common histology (15, 55.6%). Median age was 67 years, and most patients were male (74.1%). Four patients (14.8%) had an ECOG performance status of 0, 14 (51.9%) were ECOG 1 and nine (33.3%) were ECOG 2. Most were advanced-stage, with stage IV NSCLC plus extensive-stage SCLC totaling 19 cases (70.4%). Out of the 21 NSCLC cases, eight did not have a molecular testing panel available at the time of treatment decision, including two cases without a definitive histologic diagnosis. Full baseline characteristics are shown in Table 1.

Across all 27 cases, appropriateness scores were high for every source. Mean scores were 4.52 for the real-world decision, 4.48 for ChatGPT, 4.44 for Claude, 4.41 for Grok and 4.37 for Gemini (Table 2). The Friedman test showed no significant difference across the five sources (χ^2^_4_ = 1.99, *p* = 0.74). No recommendation from any source received a score below 3, indicating that all were judged at least partially acceptable (a ceiling effect).

Among the 21 NSCLC cases, the 13 with molecular results available received a mean appropriateness score of 4.55 (primary reviewer, all sources), compared with 4.33 for the eight without molecular results (AI-only means 4.54 and 4.34, respectively). Recommendations were therefore rated modestly lower when molecular data were missing but remained within the acceptable range, indicating that the models handled missing biomarker information reasonably while still reflecting the genuine uncertainty it introduces.

All 135 recommendations were independently re-scored by a second reviewer (see Section 2), blinded to source and to the first reviewer’s scores. Raw agreement between the two reviewers was high: scores were identical in 61.5% of recommendations and within one point in 93.3%. Chance-corrected agreement was fair (weighted Cohen’s κ = 0.26). This low value is a direct consequence of the skewed score distribution rather than of poor concordance: because both reviewers assigned high scores to nearly all recommendations, chance-expected agreement was already 52%, and the maximum weighted κ attainable given the two reviewers’ marginal distributions was only 0.52. The observed κ thus represents roughly half of the maximum achievable under these marginals—a recognized behavior of κ under skewed distributions (the “kappa paradox”) [17]. The second reviewer scored slightly more leniently (mean 4.82 vs. 4.44), but the two disagreed by more than one point in fewer than 7% of cases.

The primary reviewer flagged seven recommendations as potentially unsafe (Table 2: one real-world, two ChatGPT, two Claude, two Grok, zero Gemini), whereas the second reviewer flagged one. Agreement between reviewers on this flag was high in raw terms (95.6%), though κ was low (0.24), again reflecting the rarity of the flag and the same skew that attenuated the score κ. Notably, the single recommendation that both reviewers independently flagged as potentially unsafe—and the only real-world recommendation either reviewer scored below 4—was a real-world treatment decision, not an AI output. Inter-rater agreement for the guideline-concordance and local/supportive-omission flags was likewise high in raw terms (80.0% and 97.8%, respectively) but limited by marked skew. Quality flags were otherwise broadly similar across sources (Table 2): recommendations were rated guideline-concordant in 67–89% of cases, with the real-world decision and ChatGPT highest (both 89%) and Gemini lowest (67%). Gemini’s lower concordance flag rate despite a mean appropriateness score within the range of the other sources indicates recommendations that were generally acceptable but more often diverged from the specific guideline-preferred option; with a single query per model and only 27 cases, a few divergent outputs shift this proportion considerably, so the spread should be interpreted cautiously. Potentially unsafe flags were rare (0–7%), as were omissions of local or supportive treatment (0–4%). Given the small sample, flags were not tested statistically.

In a sensitivity analysis stratified by reviewer, the second reviewer’s mean scores by source were 4.78 (real-world), 4.89 (ChatGPT), 4.85 (Claude), 4.85 (Gemini) and 4.74 (Grok). Although systematically higher than the primary reviewer’s, these scores showed the same absence of a significant between-source difference (Friedman χ^2^_4_ = 4.00, *p* = 0.41). The null result was therefore independent of which reviewer’s scores were analyzed, indicating that the conclusion does not depend on rater stringency (Supplementary Table S1).

Recommendation length varied by source, from a mean of 80 words (Grok) to 126 words (Claude), with two models exceeding the 120-word cap. Length did not predict appropriateness (Spearman ρ = −0.01, *p* = 0.91); concise and verbose recommendations frequently received the same score, consistent with the reviewer grading clinical content rather than rewarding longer responses. The full set of 135 recommendations is available (see Data Availability) for readers wishing to compare outputs directly.

## 4. Discussion

In this single-center feasibility study, AI-generated treatment recommendations for newly diagnosed lung cancer received appropriateness scores similar to those given to the treatment patients actually received, with no statistically significant difference detected across the four models and the real-world decision. Scores were high throughout, and no recommendation was scored below the midpoint of the scale. These findings are consistent with a growing literature suggesting that current LLMs can produce treatment recommendations broadly aligned with expert decisions in oncology [4,8,9] while also underscoring the well-documented caveats around the reliability and reproducibility of consumer chatbots in clinical use [18]. Two caveats temper this, however. First, scores clustered near the top of the scale for every source, including the real-world decision, so the study had little room to discriminate between them—a ceiling effect that limits what the null result can tell us. Second, and importantly, the absence of a detected difference is not evidence of equivalence: with 27 cases, the study was underpowered to detect anything but large differences, and the results should be read as hypothesis-generating rather than reassuring. With scores compressed into the upper third of the scale, the small between-source differences should not be read as a ranking, and a larger, more varied cohort might reveal separation that the present design could not. It is also notable that the one recommendation both reviewers independently flagged as potentially unsafe was a real-world decision rather than an AI output; we draw no strong inference from a single case, but it is a useful corrective to the assumption that the human decision is necessarily the safe reference. Notably, recommendation length did not predict appropriateness, suggesting the reviewer graded clinical content rather than rewarding longer or more elaborate responses.

Recent studies have compared LLM recommendations with multidisciplinary decisions in oncology in melanoma [5], primary liver tumors [6], and breast cancer [7], with concordance varying by model and setting. Our findings extend this work to lung cancer, a setting where treatment depends heavily on stage, histology and molecular profile. They also differ in one respect: in those studies, the multidisciplinary decision served as the reference standard against which the models were judged, whereas here all five recommendations, including the real-world decision, were scored on the same scale by two reviewers blinded to the source. The human comparator was therefore subject to the same scrutiny as the models rather than assumed correct, which may explain why it did not stand clearly apart from them.

## 5. Limitations

This study has several limitations. The most important limitation concerns the primary reviewer (MJ-P), who is a co-author and the spouse of the corresponding author. Two features of the design constrain, but do not eliminate, the resulting risk of bias. First, she was blinded to the source of every recommendation and could not know which of the five was the real-world decision or which model produced each AI recommendation. Second, she was nonetheless aware that each case contained exactly one real-world decision among the five—a partial un-blinding that could, in principle, have biased scoring for or against the human arm. To bound this, a fully independent second reviewer (CȘS) re-scored all 135 recommendations under identical blinding; his scores were systematically more lenient but produced the same null result across sources, indicating that the central finding does not rest on the primary reviewer’s judgments. The two reviewers practice at different institutions, strengthening the independence of the reliability assessment; however, the second reviewer shares an institution with the source cases, so residual familiarity with local prescribing patterns cannot be fully excluded. We judged transparent disclosure of this relationship preferable to any arrangement that obscured it, and it is disclosed here, in the Conflicts of Interest statement, and in the author contributions. Agreement between the two reviewers was high in raw terms (93.3% within one point) but only fair after chance correction (weighted κ = 0.26), a value substantially suppressed by the ceiling effect described above.

The cohort also limits generalizability in two related ways. It was weighted toward advanced disease (70.4% stage IV NSCLC or extensive-stage SCLC), where systemic therapy is frequently the standard backbone and the decision space is correspondingly narrower; potentially resectable stage I–III disease, in which operability, the choice and extent of resection, nodal staging, and the sequencing of neoadjuvant and adjuvant therapy drive considerably more nuanced decisions, was underrepresented. The high appropriateness scores may therefore partly reflect this case mix, and the findings should not be extrapolated to early-stage or surgically managed disease. In addition, the cohort was relatively fit (no patient had ECOG performance status 3–4); fitter patients tend to present more guideline-canonical decisions with fewer trade-offs around tolerability and best supportive care, which may have further contributed to the uniformly high scores and limits generalizability to frailer populations. The sample was small (27 cases), single-center, and specific to one Romanian outpatient department, and the study was underpowered to detect any but large differences.

Several interface-level factors limit the reproducibility of our findings. All models were queried through consumer web interfaces rather than version-pinned application programming interfaces (APIs), so generation parameters (e.g., temperature) could not be fixed, and providers may alter the underlying models without notice; one interface (Grok) did not display a model version identifier at all. In addition, minor procedural anomalies occurred: a personalization setting affecting response style was inadvertently active for the initial ChatGPT queries (through case LCAI-18), and one Claude case was entered in an existing rather than a new session. Although the clinical content of the affected outputs was reviewed and judged unaffected, factors such as active personalization, session carryover, and unpinned model versions can influence output consistency and cannot be fully excluded as sources of variation. Each case was queried once, so intra-model response variability was not assessed. Future studies should query models through version-pinned API access with fixed generation parameters and isolated sessions to ensure parameter stability and full reproducibility.

Importantly, the human comparator was not a prospectively recorded multidisciplinary tumor board recommendation. In this setting, multidisciplinary discussion is not formally documented as a structured decision, so the comparator was reconstructed by the senior oncologist from the treatment actually delivered and from routine clinical documentation rather than from a recorded MDT consensus. It should therefore be understood as AI versus real-world clinical practice, not AI versus a formal tumor board; we have avoided describing it as an “MDT decision” except when referring to how such decisions are ordinarily reached. Moreover, the human comparator was reconstructed post hoc into the study’s output format. Fields concerning prospective uncertainty (acceptable alternatives, missing information, and major concern) are inherently difficult to populate retrospectively, which may have systematically disadvantaged the human comparator relative to the models; conversely, the reconstruction was performed after the model outputs were available, which may have had the opposite effect. The net direction of this asymmetry is unknown. Prospective designs that capture the MDT decision in real time, such as Dogan et al. [19], avoid this asymmetry and represent the preferred approach for future studies.

## 6. Implications and Future Directions

Beyond the specific comparison, this study shows that a blinded, structured evaluation of AI-generated recommendations against real-world care is feasible in a routine clinical setting, using only anonymized case summaries and a single standardized prompt. A scaled version would address the main limitations directly: a larger, ideally multi-center cohort with a broader case mix, several independent blinded reviewers to allow assessment of inter-rater reliability, and prospective capture of the treatment decision before model outputs are generated. Such a design would move beyond feasibility toward a genuine test of whether AI recommendations can safely support multidisciplinary decision-making in lung cancer. The real-world decision was itself judged guideline-concordant in only 89% of cases, reflecting the practice variability expected where multidisciplinary input and molecular testing are not uniformly available. In such resource-limited settings, a validated, guideline-anchored AI tool could in principle serve as a low-cost second opinion—flagging decisions that diverge from current standards before treatment is initiated—provided its recommendations are first shown to be safe and reliable in adequately powered, prospective studies [11]. The feasibility framework demonstrated here is a first step toward enabling smaller centers to perform that validation locally.

## 7. Conclusions

In this feasibility study, appropriateness scores were high and similar across AI models and real-world decisions, clustering near the top of the scale. The small sample and ceiling effect preclude conclusions about equivalence; the findings are hypothesis-generating and establish the feasibility of this evaluation method. These results reflect specific outputs generated by particular consumer interfaces on defined dates in June 2026, not stable or standardized characteristics of the named AI platforms, which their providers may alter without notice, and should be read accordingly. Confirmation in larger, multi-center studies with multiple independent reviewers is warranted.

## Figures and Tables

**Table 1:** Baseline characteristics (N = 27).

Characteristic	n (%) or Median [Range]
Age, years, median [range] ^a^	67 [40–75]
Sex	
Male	20 (74)
Female	7 (26)
Smoking status	
Current	9 (33)
Former	5 (19)
Never	3 (11)
Unknown	10 (37)
ECOG performance status	
0	4 (15)
1	14 (52)
2	9 (33)
Cancer type	
Non-small-cell	21 (78)
Small-cell	6 (22)
Histology	
Adenocarcinoma	15 (56)
Squamous cell carcinoma	4 (15)
Small-cell carcinoma	6 (22)
Unknown/pending	2 (7)
Stage	
II	1 (4)
III	6 (22)
IV (NSCLC)	14 (52)
Limited-stage (SCLC)	1 (4)
Extensive-stage (SCLC)	5 (19)
Advanced (stage IV NSCLC + ES-SCLC)	19 (70)
Biomarkers	
Actionable driver detected ^b^	6 (22)
PD-L1 ≥ 50%	3 (11)
No molecular result available (NSCLC)	8/21 (38)
Prior treatment	
Prior surgery ^c^	6 (22)
Prior radiotherapy	2 (7)
Prior systemic therapy	0 (0)

^a^ Documented in 25/27 cases. ^b^ EGFR (n = 3), ALK (n = 1), BRAF (n = 1), and MET exon 14 (n = 1). ^c^ Heterogeneous: diagnostic or incomplete resection or prior brain surgery. Full baseline characteristics, including organ function, individual metastatic sites, and access/reimbursement context, are included in File S2.

**Table 2:** Appropriateness scores and quality flags by source (N = 27 per source).

Source	Mean Score	Median	Score ≥ 4, n (%)	Guideline-Concordant, n (%)	Potentially Unsafe, n (%)	Local/Supportive Omitted, n (%)
Real-world	4.52	5	25 (93)	24 (89)	1 (4)	0 (0)
ChatGPT	4.48	5	24 (89)	24 (89)	2 (7)	0 (0)
Claude	4.44	5	23 (85)	21 (78)	2 (7)	1 (4)
Grok	4.41	5	24 (89)	20 (74)	2 (7)	1 (4)
Gemini	4.37	5	23 (85)	18 (67)	0 (0)	1 (4)

Friedman χ^2^_4_ = 1.99, *p* = 0.74 (appropriateness scores; no significant difference across sources). All scores ranged 3–5 (no recommendation scored below 3).

## Data Availability

The following Supplementary Materials are available from the corresponding author on reasonable request: the abstraction form, the complete list of recommendations (135), one example case batch (containing the first case summary file, the instructions for the reviewers and the score cards), and the randomization table/blinding key.

## Supplementary Materials

The following supporting information can be downloaded at https://www.mdpi.com/article/10.3390/curroncol33100640/s1: File S1: Standardized Prompt; File S2: Anonymized Study Dataset (case characteristics and both reviewers’ scores); File S3: CHART Reporting Checklist; File S4: R Statistical Analysis Code; Table S1: Appropriateness scores by source for both reviewers.
