# Supplementary material for: AI-Generated Treatment Recommendations in Newly Diagnosed Lung Cancer: A Single-Center Feasibility Study in a Resource-Limited Setting
Source: Curr Oncol. 2026 Oct 21;33(10):640. doi: 10.3390/curroncol33100640 (PMC13703460; doi:10.3390/curroncol33100640)
Supplement: Supplementary file 1 [file curroncol-33-00640-s001.zip › 05_Table_S1_scores_by_reviewer.pdf]

Table S1. Appropriateness scores by source for both reviewers (N = 27 per source).

Source	Reviewer 1 mean	Reviewer 1 median	Reviewer 2 mean	Reviewer 2 median
**Real-world**	4.52	5	4.78	5
**ChatGPT**	4.48	5	4.89	5
**Claude**	4.44	5	4.85	5
**Gemini**	4.37	5	4.85	5
**Grok**	4.41	5	4.74	5

Friedman rank-sum test across the five sources: Reviewer 1 χ^2_4 = 1.99, P = .74; Reviewer 2 χ^2_4 = 4.00, P = .41. Neither reviewer showed a significant between-source difference. Overall mean score: Reviewer 1, 4.44; Reviewer 2, 4.82. Weighted Cohen's κ (quadratic) between reviewers = 0.26; exact agreement 61.5%, within-one-point agreement 93.3%.
