# Supplementary material for: AI-Generated Treatment Recommendations in Newly Diagnosed Lung Cancer: A Single-Center Feasibility Study in a Resource-Limited Setting
Source: Curr Oncol. 2026 Oct 21;33(10):640. doi: 10.3390/curroncol33100640 (PMC13703460; doi:10.3390/curroncol33100640)
Supplement: Supplementary file 1 [file curroncol-33-00640-s001.zip › File S1 01_Prompt.pdf]

You are given a structured lung cancer case summary (attached file).

Task:
Based only on the information provided, recommend the best initial treatment decision for this lung cancer patient, according to NCCN-style lung cancer management principles. Include both:
1. Systemic treatment, if appropriate.
2. Local treatment or supportive interventions, if relevant, including brain radiotherapy, surgery, palliative radiotherapy, bone-directed treatment, symptom control, or urgent stabilization.
Do not limit the answer to systemic therapy only.
If a local treatment decision depends on missing details, state what is missing.

Rules:
- Do not invent missing information.
- If essential information is missing, state it.
- Keep the answer concise.
- Use only the required format.
- Maximum 120 words total. If essential missing information must be listed, prioritize clinical safety over word count.
- Do not include generic disclaimers.
- Do not mention that you are an AI.
- Do not list as an acceptable alternative any treatment that is contraindicated or relatively contraindicated based on the case details. If relevant, mention such treatment only under Major concern as a treatment to avoid.
- For local/supportive treatment, recommend only interventions supported by the symptoms, imaging, or risk features provided. Do not assume symptoms are present unless stated.

Specificity rule:
When sufficient information is available, recommend a specific treatment regimen, including drug names where appropriate.
Avoid vague class-only answers such as “platinum doublet” unless the available data are insufficient to choose a specific regimen.
If several guideline-acceptable options exist, name one preferred option and briefly list acceptable alternatives.
If missing information prevents choosing between options, state the missing information and give a conditional recommendation.

Strictly respect this format:
Treatment intent:

Preferred systemic treatment: [Name specific regimen if possible. If not possible, explain what missing information prevents specificity.]
Local / supportive treatment: [Brain RT, palliative RT, surgery, bone-directed treatment, supportive care, etc., if relevant.]
Acceptable alternatives: [List only clinically reasonable alternatives.]
Missing information that could change decision:
Key reason:
Major concern:

[CASE TO BE ATTACHED AS docx file]
